# What is the effect of bariatric surgery on health-related quality of life in people with obesity? Observational cohort analysis of the United Kingdom National Bariatric Surgery Registry

**DOI:** 10.1097/JS9.0000000000002044

**Published:** 2024-08-22

**Authors:** John Buckell, Peter K. Small, NBSR Collaborators, Susan A. Jebb, Paul Aveyard, Omar Khan, Emma Rose McGlone

**Affiliations:** a Health Economics Research Centre, Oxford Population Health, University of Oxford; b Department of Bariatric and Upper Gastro-Intestinal Surgery, Sunderland NHS Foundation Trust; c British Obesity and Metabolic Surgery Society (BOMSS); d Nuffield Department of Primary Health Care Sciences, University of Oxford; e Department of Bariatric and Upper Gastro-Intestinal Surgery, St George’s University Hospitals NHS Foundation Trust, St George’s University of London; f Department of Surgery and Cancer, Imperial College London, Faculty of Medicine, London, UK

**Keywords:** bariatric surgery, health care economics, health-related quality of life, obesity, type 2 diabetes

## Abstract

**Background:**

Previous small studies investigating health-related quality of life (HRQoL) following bariatric surgery have demonstrated heterogenous effects. This study aimed to use National Bariatric Surgery Registry (NBSR) records to investigate the relationship between weight and HRQoL in people undergoing bariatric surgery in the UK.

**Materials and methods:**

In this observational study using United Kingdom National Bariatric Surgery Registry (NBSR) records between 1st June 2017 and 23rd November 2022, patients undergoing primary bariatric surgery with one baseline and at least one follow-up visit within 1 year from surgery were eligible for inclusion. Models estimated the relationship between EuroQol Five Dimension 5-level (EQ-5D) and BMI at baseline and longitudinally. Further analyses were stratified by type 2 diabetes, type of operation (adjustable gastric band, sleeve, or bypass), and domain of EQ-5D.

**Results:**

Five thousand five hundred eighty-seven observations of 2160 patients were analysed. At baseline, the mean BMI was 45.7±7.8 kg/m^2^ and the mean EQ-5D was 0.78±0.22. A 1 kg/m^2^ higher BMI was associated with 0.005 (95% CI [−0.006 to −0.004]) lower EQ-5D. In the month following surgery, EQ-5D increased to 0.91±0.2 while BMI decreased to 39.8±7.1 kg/m^2^ (*P*<0.001 for both); subsequently, EQ-5D plateaued (0.90±0.17 at 12 months) while BMI continued to decrease (31.5±6.2 kg/m^2^ at 12 months, *P*<0.001). Each 1 kg/m^2^ decrease in BMI was associated with a 0.006 (95% CI [−0.007 to −0.005]) increase in EQ-5D. Remission of T2D was independently associated with increase in EQ-5D (0.037, 95% CI [0.015–0.059]); type of operation was not. Decreases in BMI were associated with improvements in all five domains of EQ-5D.

**Conclusions:**

In this large dataset, greater weight loss and T2D remission were independently associated with greater improvements in HRQoL following bariatric surgery. The HRQoL-BMI relationship for people undergoing bariatric surgery differs to that which has previously been estimated following behavioural interventions. The use of the estimates generated here will be important for clinical and political decision-making.

## Introduction

Highlights

- To date, the relationship between health-related quality of life (HRQoL) and weight has not been estimated for people undergoing bariatric surgery.
- Using a national registry dataset we quantified the effect of bariatric surgery on HRQoL.
- Greater weight loss is independently associated with greater improvements in HRQoL.
- Remission from type 2 diabetes, but not type of operation, is independently associated with greater improvements in HRQoL.

Obesity is estimated to cause 4.7 million excess deaths annually, disproportionately affecting people experiencing socio-economic deprivation^1^. Type 2 diabetes, which is strongly associated with obesity, is one of the leading causes of global health burden attributable to high BMI^1,2^. Along with complex physical and psychosocial comorbidities^3,4^, people living with obesity have impaired generic and obesity-specific health-related quality of life (HRQoL)^5–7^, defined as ‘a multidomain concept that represents the patient’s general perception of the impact of an illness and its treatment on physical, psychosocial, and social aspects of life’^8^.

Bariatric surgery is an effective treatment for obesity, resulting in marked and sustained weight loss^9^, resolution or improvement of type 2 diabetes^10^, and reduced psychosocial distress^4^. There is limited evidence, however, regarding the impact of bariatric surgery on HRQoL. It is likely that many aspects of HRQoL improve following surgery^11^; however, estimating the size of the effect is difficult due to the lack of standardized measures of HRQoL, poor study design, and small numbers of patients^8,12,13^. A systematic review of existing studies suggests that effects may be greater for physical domains of HRQoL than mental domains^8,14^.

Estimated changes in HRQoL are used to evaluate both clinical-effectiveness and cost-effectiveness of interventions^15,16^. These analyses, in turn, inform commissioning decisions by bodies such as the National Institute of Health and Care Excellence (NICE), which determines national healthcare resource allocation in the UK^17^. Understanding the HRQoL of patients undergoing bariatric surgery is therefore important for both clinical practice and policymaking. Inaccurate estimates of the relationship between HRQoL and BMI can lead to errant decision-making^6^. To date, the HRQoL-BMI relationship has not been estimated for people undergoing bariatric surgery. Given that these patients typically have higher baseline BMI and lose more weight than patients receiving behavioural interventions, it is important to assess the relationships between BMI, weight change, and HRQoL in this specific population.

The National Bariatric Surgical Registry (NBSR) is a national database of prospectively-collected data for patients undergoing bariatric surgery in the UK. The aim of this study was to use this large observational dataset to examine the relationship between HRQoL and BMI in patients undergoing primary bariatric surgery.

## Methods

### Study design and setting

This was a retrospective analysis of prospectively-collected data from the NBSR, an anonymised, bespoke record of bariatric cases carried out within the National Health Service (NHS) in the UK and Ireland^18^. Our analysis plan was prospectively published in March 2022, and it is available here: https://osf.io/6t9rg/. It has been retrospectively registered at on ClinicalTrials.gov with the identifier NCT06324526 and can be accessed at https://clinicaltrials.gov. This study was conducted according to the principles of the Declaration of Helsinki^19^. The data holder NBSR complied with local ethics guidelines and use of this dataset for research purposes conformed with UK legislation and was approved by the Health Research Authority (17/CAG/0023). The study has been reported as per the strengthening the reporting of cohort, cross-sectional, and case–control studies in surgery (STROCSS) checklist for observational studies^20^.

Completion of the NBSR registry is mandated by NHS England. NBSR data are recorded by clinicians responsible for the patients and include baseline and follow-up demographic and clinical variables. EQ-5D is collected as a measure of HRQoL at baseline and follow-up visits. HRQoL data has been collected in the NBSR since 1st June 2017, and we studied records from this date to 23rd November 2022.

### Patients

All adults (18 years or older) undergoing primary bariatric surgery in the UK were eligible for inclusion. This is available to people that fulfil NICE criteria for surgery, which during the period studied included BMI of 40 or more, or a BMI between 35 and 40 and an obesity-related condition (such as type 2 diabetes or high blood pressure)^21^. We excluded patients undergoing revisional surgery and censored any follow-up after revisional surgery. We selected only records that included a complete EQ-5D scoring (all five domains). Since our focus was the effect of bariatric surgery on HRQoL over time from surgery, we retained only patients with both baseline (preoperative) and at least one follow-up record. We excluded records with implausible values, defined as BMI <25 kg/m^2^ or >100 kg/m^2^; height <1 m or >2.5 m; weight <50 kg or >400 kg; age >100 years. Additionally, we excluded follow-up records greater than 12 months from the surgery date due to limited records. Each patient was seen once at baseline, and then one or more follow-up visit(s) over the next 12 months (which could have occurred on any of the 12 months following baseline).

### Variables

#### Main outcome and exposure

The main outcome was EuroQol Five Dimension 5-level (EQ-5D^22^), a general population, preference-based health status measure. Although there are many validated measures for HRQoL^8^, EQ-5D is recommended by NICE^23^ and has good validity in people with type 2 diabetes^24^. Subjects are asked to rate the level of problems they experience related to five domains of HRQoL (mobility, self-care, usual activities, pain/discomfort, and anxiety/depression), with a lower score indicating better quality of life. EQ-5D scores are converted to overall utilities by averaging domains and weighting by the general public’s valuation of the domains^25^. A higher overall utility for EQ-5D represents better HRQoL. The main explanatory variable was BMI. We planned subgroup analysis by type 2 diabetes status, type of operation (adjustable gastric band, sleeve, or bypass), and domain of EQ-5D.

#### Control variables

We included binary variables for female (reference: male) and non-white ethnicity (reference: white). A categorical variable for employment included employed, unemployed, retired, and not reported. Age was captured continuously and specified linearly in models. We included a binary variable for type 2 diabetes (reference: no type 2 diabetes) with presence of type 2 diabetes defined as taking any type 2 diabetes medications. Remission of type 2 diabetes was defined as discontinuation of all type 2 diabetes medications. We also included a categorical variable of 0-5 comorbidities (hypertension, sleep apnoea, musculoskeletal pain, gastro-oesophageal reflux disease, and metabolic liver disease). All comorbidities were defined as either absent, or present, on or off treatment. We also included a categorical variable for types of surgery (band, bypass, and sleeve). For simplicity, we pooled Roux-en-Y and single-anastomosis procedures in the bypass category. Hospital fixed effects capture hospital-specific, individual-invariant variation in HRQoL.

### Statistical analysis

Statistical analysis was performed using R, with the package ‘mgcv’ version 1.9-1^26^. Descriptive statistics are presented at baseline and presented as mean with SD or median and interquartile range, for all observations and partitioned by type 2 diabetes status and operation type. Change in HRQoL and BMI over time-from-operation was analysed by between period *t*-tests.

Linear models regressed HRQoL (EQ-5D) on BMI and the control variables. A first regression modelled baseline HRQoL on baseline BMI in a cross-sectional analysis (model 1). We then examined the change in EQ-5D as a function of change in BMI (model 2). Individual random effects controlled for individual-specific, time-invariant variation in HRQoL. Group means variables of BMI were entered into the regression to obtain within-estimates of the HRQoL-BMI relationship^27^. We specified natural splines on calendar time to capture the average variation in HRQoL over time. Time-from-operation, which varies independently of calendar time because patients’ dates of operation vary, was specified linearly capturing average variation in HRQoL from baseline.

We analysed how the change in HRQoL related to the change in BMI by its constituent domains. The model specification is as above except that the dependent variables are the changes in the domain score rather than the changes in overall EQ-5D.

All models were fitted in R (version 4.2.3). Linear models used the package *lme4*. Domain-specific models treated each response as an ordered variable. These models used generalised additive ordered categorical regressions using the package *mgcv*. Here, the linear predictor is a latent variable with estimated thresholds that mark the transitions between levels of the ordered categorical response. The package *splines* was used to generate splines in all models.

Sensitivity analyses were conducted as follows: comparison of individuals that were observed only once in the sample to those from a sample that included individuals observed more than once; several specifications of splines (including treating variables linearly) for continuous models; dummy-coded, rather than continuous, BMI, classified as healthy weight, overweight, and classes 1 to 3 obesity. In an ad-hoc sensitivity analysis, we used binary variables of GORD remission, GORD development and postsurgery complications to capture any independent association with HRQoL.

## Results

### Study records

There were 7537 people recorded as having primary bariatric surgery from 1st July 2017 onwards (Supplementary Figure 1, Supplemental Digital Content 1, http://links.lww.com/JS9/D356). Baseline records were merged with follow-up records, giving 20 304 records in total. Six thousand five hundred fifty-four records were excluded because EQ-5D data were missing or incomplete; a further 289 records were excluded because type 2 diabetes status was missing. For comorbidities apart from type 2 diabetes the missing data rate was less than 1%: where information was lacking the patient was assumed not to suffer from the condition. For all other variables the records were 100% complete. There were few records occurring more than 12 months after surgery (*n*=1164) and these were excluded. The distribution of other records over time is shown in Supplementary Table 1 (Supplemental Digital Content 1, http://links.lww.com/JS9/D356). Four thousand seven hundred fifty-nine records were excluded because they referred to a patient for whom there was no follow-up record available within the 12-month study timeframe. Our final dataset for analysis comprised 5587 records for 2160 patients.

Patients included in the study were predominantly female (85%) and white (90%). The majority underwent sleeve gastrectomy (53%), a smaller proportion underwent Roux-en-Y gastric bypass/single-anastomosis gastric bypass (‘gastric bypass’; 42%), and very few had an adjustable gastric band (4.8%). In patients with type 2 diabetes (*n*=331, 15%), the proportion undergoing adjustable gastric band was even lower (1.5%) and most patients underwent gastric bypass. Table 1 gives descriptive statistics of patient characteristics.

**Table 1:** Baseline characteristics of patients included in the study.

	Total cohort	Type 2 diabetes status		Type of operation		
		No	Yes	AGB	SG	GB
		*N*=1829	*N*=331	*N*=103	*N*=1146	*N*=911
	*N*=2160	(85%)	(15%)	(4.8%)	(53%)	(42%)
Mean EQ-5D (SD)	0.78 (0.22)	0.79 (0.21)	0.70 (0.24)	0.82 (0.22)	0.81 (0.22)	0.73 (0.21)
Mean BMI kg/m^2^ (SD)	45.7 (7.8)	45.6 (7.8)	46.5 (8.0)	41.6 (7.54)	45.5 (8.0)	46.6 (7.4)
Mean age years (SD)	43.6 (11.3)	42.5 (11.1)	49.6 (10.3)	43.5 (11.4)	42.3 (11.5)	45.3 (10.8)
Female (%)	85	87	72	86	87	83
White (%)	90	90	89	85	88	92
Index of multiple deprivation (centile)	51.6 (29.3)	52.1 (29.5)	48.9 (27.9)	51.3 (32.1)	51.6 (29.8)	51.7 (28.3)
Median comorbidity count (IQR)	1 (2)	1 (1)	2 (2)	1 (0)	0 (2)	1 (2)
Operation n (%)						
AGB: SG: GB	103 (4.8): 1146 (53): 911 (42)	98 (5.4): 1042 (57.0): 689 (37.7)	5 (1.5): 104 (31.4): 222 (67.0)	–	–	–
Type 2 Diabetes, *n* (%)	331 (15)	–	–	5 (5)	104 (9)	222 (20)

AGB indicates Adjustable gastric band; GB, gastric bypass and single-anastomosis gastric bypass; IQR, interquartile range; SD, standard deviation; SG, Roux-en-Y sleeve gastrectomy.

### Relationship between HRQoL and BMI at baseline

**Figure 1:**
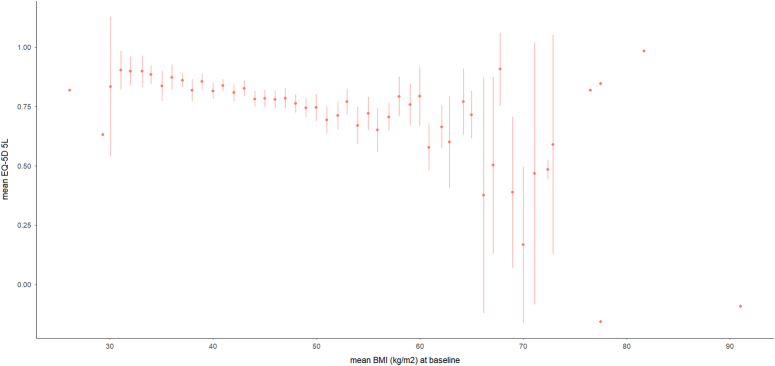
Association between BMI and EQ-5D. *n*=2160. Mean and 95% CI.

At baseline, the mean BMI was 45.7±7.8 kg/m^2^ and the mean EQ-5D was 0.78±0.22. Higher BMI was associated with a lower EQ-5D score (Fig. 1). Patients with type 2 diabetes had lower EQ-5D scores compared to those without (Table 1). Patients who subsequently underwent gastric bypass had lower EQ-5D scores than those who underwent adjustable gastric band or sleeve gastrectomy (Table 1).

At baseline, domain scores were as follows: mobility 0.77 (0.74–0.80), self-care 0.86 (0.84–0.89), usual activities 0.83 (0.80–0.86), pain/discomfort 0.79 (0.76–0.81), and anxiety/depression 0.81 (0.75–0.85).

After adjustment for other variables, at baseline, a greater BMI was associated with lower EQ-5D (est=−0.005; 95% CI [−0.006 to −0.004] per kg/m^2^; Supplementary Table 2, Supplemental Digital Content 1, http://links.lww.com/JS9/D356, Model 1). Female sex (male vs. female) est=0.031; 95% CI [0.012–0.051]), being retired (est=−0.066; 95% CI [−0.103 to −0.030]) or unemployed (est=−0.090; 95% CI [−0.111 to −0.069]) versus being employed, increasing number of comorbidities (all negative and statistically significant for 1 to 4 comorbidities versus none) and older age (est=−0.001; 95% CI [−0.002 to 0.000]) per year were also associated with lower EQ-5D score. Type 2 diabetes was not associated with EQ-5D at baseline and neither was EQ-5D associated with the type of operation selected.

### Longitudinal relationship between HRQoL and BMI

**Figure 2:**
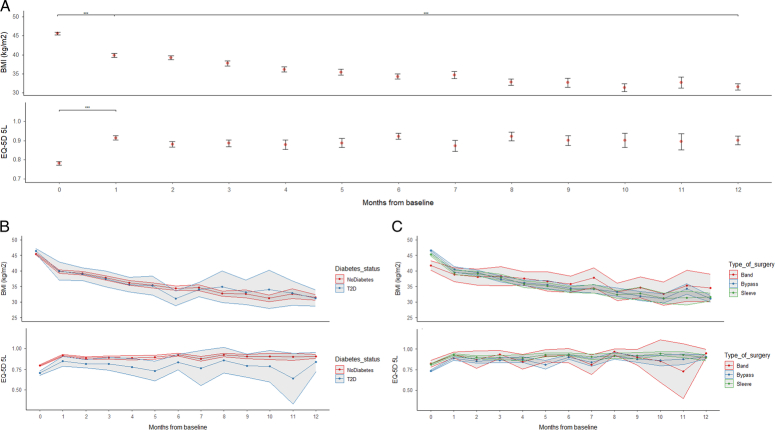
Association between BMI and EQ-5D over 12 months from baseline. Panel A, whole population (with 95% CI; ***=*P*<0.001 between periods). Panel B, by type 2 diabetes status. Panel C, by type of surgery.

BMI decreased and EQ-5D increased in the first month following surgery to 39.8±7.1 kg/m^2^ and 0.91±0.2, respectively (*P*<0.001 for both); following this BMI continued to fall to 31.5±6.2 kg/m^2^ at 12 months, whereas EQ-5D plateaued (0.90±0.17 at 12 months) (Fig. 2, Panel A).

Factors that were independently associated with improved EQ-5D (Supplementary Table 2, Supplemental Digital Content 1, http://links.lww.com/JS9/D356, Model 2) included greater reduction in BMI, with a 1 kg/m^2^ decrease associated with a 0.006; 95% CI [−0.007 to −0.005] increase, and remission from type 2 diabetes, which was associated with a 0.037; 95% CI [0.015–0.059] increase. Other factors were being retired (est=0.028; 95% CI [0.000–0.055]) or unemployed (est=0.019; 95% CI [0.003–0.035]). Type of operation was not independently associated with change in EQ-5D following surgery.

### Longitudinal relationship between HRQoL and BMI by domain of HRQoL

Table 2 shows the estimated coefficients for the domain-specific analysis. For every domain, an increase in BMI was associated with an increase in reported score. Given that a lower score represents a better HRQoL, the patterns are consistent with the increases in the overall EQ-5D index observed following a reduction in BMI. The magnitudes suggest that gains were realised most in mobility and pain/discomfort, and least in self-care.

**Table 2:** Domain-specific analysis of estimated EQ-5D change for each unit BMI change.

	Estimate	Std. error	LCB	UCB	*t*-value	Pr(>|t|)
Mobility	0.025	0.002	0.020	0.029	10.739	<0.001
Self-care	0.014	0.002	0.011	0.017	8.750	<0.001
Usual activities	0.021	0.002	0.017	0.025	10.095	<0.001
Pain and discomfort	0.025	0.003	0.020	0.030	9.920	<0.001
Anxiety and depression	0.022	0.003	0.017	0.027	8.852	<0.001

Std. Error - robust standard error, Inds – number of individuals, Obs – number of observations, LCB – lower bound of 95% CI, UCB – upper bound of 95% CI, *t*-value – t-ratio, P(>|t|) – probability of being greater than the absolute critical value of t, AIC – Akaike Information Criterion. Estimates show the association of a one-unit change of BMI with the change of the domain of EQ-5D.

### Sensitivity analyses

We included individuals that were observed only once in the sample and compared cross-sectional results to those from the sample that included individuals observed more than once. We used several model specifications to assess the robustness of the relationship to those specifications. We tested several specifications of splines (including treating variables linearly) for continuous models, and retained models with splines that yielded the best fit of the data (according to Akaike Information Criteria). An ad hoc analysis of privately funded patients neither was significant as a covariate nor impacted on the main findings. The estimates were stable in all cases. Estimates were robust to the inclusion of the GORD and postsurgery complications variables.

## Discussion

In this nationwide, real-world registry analysis of over 5000 records from more than 2000 patients, we demonstrate that higher BMI is associated with lower HRQoL in people prior to bariatric surgery. Weight loss following bariatric surgery independently predicts improvements in HRQoL. This was found across all domains of HRQoL assessed by EQ-5D. Greater improvement in HRQoL was independently associated with remission of type 2 diabetes but not operation type.

Our finding that bariatric surgery is associated with improved HRQoL corroborates data from systematic reviews and meta-analysis^14,28–30^. These previous studies have, however, reported considerable heterogeneity in the effect size. In this population, we estimate an increase in EQ-5D of around 0.006 for each point BMI decrease; this is around half of the change that has been estimated following behavioural interventions. A possible explanation for this difference is that weight loss following dietary intervention is more reliant on individual determination and motivation, and there is also a different typical trajectory of weight loss (usually less profound and much slower with diet than after surgery). There may also be methodological differences since other studies used a three-level EQ-5D, whereas this study used a five-level EQ-5D^6^. Nonetheless, the differences highlight the importance of using HRQoL estimates specific to bariatric surgery when assessing the effectiveness of this type of treatment for obesity^16^. The National Institute of Health and Care Excellence (NICE) uses the in-depth review of changing evidence to make policy recommendations for the treatment of obesity in the UK^21^. The American Society for Metabolic and Bariatric Surgery (ASMBS) and International Federation for the Surgery of Obesity and Metabolic Disorders (IFSO) also specifically cite improved quality of life as a rationale for their strong recommendation for bariatric surgery in people with BMI ≥35 kg/m^2^, in recently updated guidelines^31^. Use of specific HRQoL estimates for people undergoing bariatric surgery, as generated by the present study, will improve clinical, economic and political decision-making.

In comparison to our findings, the recent By-Band-Sleeve randomized controlled trial of patients undergoing bariatric surgery in the UK reported a lower mean baseline EQ-5D score (0.61 versus 0.78) in people with comparable mean BMI (46.4 kg/m^2^ versus 45.7 kg/m^2^)^32^. A possible explanation for the difference is that people recruited for surgery as part of a randomized controlled trial are subject to different selection pressures than those undergoing routine NHS care; for example, rates of type 2 diabetes are lower in our study than in By-Band-Sleeve. Our baseline mean EQ-5D score is very similar, however, to that of other studies examining patients with obesity; for example a meta-analysis of randomized controlled trials of non-surgical weight loss interventions included over 10 000 patients with a mean EQ-5D of 0.79^6^; a further randomized controlled trial of 1267 adults reported a mean EQ-5D of 0.78^33^.

Although in our unadjusted data at baseline, the presence of type 2 diabetes was associated with lower HRQoL, there was no independent association, indicating that this association is likely to be due to other factors, including higher BMI. The type of operation that each patient will undergo is decided by the patient following multi-disciplinary assessment and counselling. As previously noted, we found that people who would subsequently undergo gastric bypass had poorer HRQoL at baseline^34^, but as with type 2 diabetes status, we detected no independent association. People opting for gastric bypass were heavier and more likely to have type 2 diabetes, which could explain their lower EQ-5D.

Although decreases in BMI were associated with improvements in EQ-5D, we observed a different trajectory for changes in BMI and EQ-5D over time-from-operation. Whereas EQ-5D improved very rapidly over the first month from surgery and then plateaued, BMI continued to fall. This could indicate a weight loss-independent component to improvement in HRQol for patients following bariatric surgery. Patients with obesity have a high prevalence of food addiction, which is associated with decreased quality of life and impaired emotional regulation^35^. Sweet-taste preference and food cravings decrease following RYGB, changes which are not clearly correlated with weight loss^36,37^. A possible explanation for our findings is that improved HRQoL following bariatric surgery is in part attributable to a reprieve from distressing food addiction symptoms for some patients. Additionally, especially in patients that have waited some time for their operation, having received bariatric surgery may provide an immediate sense of relief and a psychological boost^38^.

One-third to one-half of patients with type 2 diabetes experience remission following sleeve gastrectomy and gastric bypass surgery^10,39^. Here, we observed that remission of type 2 diabetes was independently associated with an improvement in HRQoL (0.037; 95% CI [0.015–0.059]). Although the effect observed was small, previous studies have demonstrated that changes in EQ-5D greater than 0.03 are clinically meaningful^6^. An improvement in HRQoL following type 2 diabetes remission could occur because of the psychological benefit of no longer feeling that a person has an illness or the reduction in medication and its adverse effects. Previous smaller studies have not shown this association^40^, or reported only a trend towards improved HRQoL in people achieving remission from type 2 diabetes after bariatric surgery^41^, but they may have been underpowered to detect this small effect.

Previous studies have suggested that bariatric surgery has a greater effect on the physical components of HRQoL than the psychological^8,14,42^. To examine this, we conducted a domain-specific analysis of the components of EQ-5D. We found evidence in keeping with prior studies wherein mobility and pain/discomfort were most improved following surgery, although all domains were positively affected. This could be explained by the observation that in our study and others^43^, mobility and pain/discomfort score were lowest at baseline, with self-care and usual activities relatively spared; potentially then, people living with obesity have more to gain in the former categories.

Strengths of this study include that it comprises a very large, prospectively-collected dataset on the experiences of patients undergoing bariatric in routine practice. A set of covariates controlled for variation, and several analyses provided consistent results. Unlike in clinical trials, an observational study has the benefit of reflecting real-world allocation to the type of surgery and is not subject to trial selection bias. It is also less likely to suffer from the Hawthorne effect – a propensity for individuals to modify their behaviour in response to an awareness of being assessed – which has been observed in self-reported HRQoL scores^44^. There are, however, some limitations. Firstly, we had to exclude many records due to missing EQ-5D data. EQ-5D completion has only relatively recently been added to the standard NBSR data collection form, thus it may be unfamiliar to some clinicians. As awareness increases regarding the importance of HRQoL outcomes following bariatric surgery, rates of completion should rise. Missing data could result in ascertainment bias and overestimation of associations if better outcomes are more likely to be reported and worse outcomes less so. However, it seems unlikely that clinicians documenting these data would be aware of whether the changes in EQ-5D were better or worse compared to all other surgeries nationwide^45^. Secondly, our follow-up was only for 1 year and future research should examine longer time periods; some smaller studies have shown a decline in HRQoL over time from surgery^46,47^ whereas others have demonstrated maintained HRQoL^34^.

There are advantages and disadvantages to the use of EQ-5D as a measure of HRQoL. Some studies support the use of EQ-5D in patients with type 2 diabetes^19^; however, the existence of multiple obesity-specific measures of HRQoL may imply that EQ-5D does not adequately capture HRQoL in this population^8^. Nonetheless, EQ-5D is the recommended HRQoL measure in the UK and it is used extensively across many studies, meaning that direct comparisons with other studies are possible, and the measure has well-understood properties. In addition, we were able to analyse EQ-5D’s constituent domains to provide further insight into the various aspects of HRQoL covered therein.

## Conclusion

In conclusion, BMI is negatively associated with HRQoL in the population undergoing bariatric surgery. Following bariatric surgery, BMI decreases and EQ-5D increases, although the trajectories differ over time. Improvements are seen in all domains of EQ-5D. Increased weight loss and remission from type 2 diabetes are independently associated with improvements in HRQoL.

## NBSR Collaborators

BOMSS NBSR Committee Members 2023: Omar Khan (Chair); Kamal Mahawar (Past Chair and Treasurer); Rachel Batterham; Christopher Pring; Waleed Al-Khyatt (Audit Subcommittee Lead); Guy Holt; Alex Miras (Research Subcommittee Lead); Andrew Currie (Consultant level, Trsut level and ICS Data Subcommittee Lead); Naveed Hossain (Trainee Representative).

PPI: Paul Chesworth; Nadya Isak.

Co-opted Members: Richard Welbourn; Ahmed Ahmed; Peter Walton; Robin Kinsman; Peter Small.

External Advisors 2022/2023: (to attend meetings) Omar Khan; Oliver Old (HQIP/CAG); Emma McGlone; Arutchelvam Vijayaraman; Michael Glaysher.

## Ethical approval

The data holder NBSR complied with local ethics guidelines and use of this dataset for research purposes conformed with UK legislation and was approved by the Health Research Authority (17/CAG/0023) on 6th April 2017.

## Consent

Patients undergoing bariatric surgery in the UK consent at the time of their operation for entry of their anonymised data to NBSR for audit and research purposes.

## Source of funding

J.B., P.A., and S.A.J. are funded by the Oxford Biomedical Research Centre. P.A. and S.A.J. are funded by the NIHR ARC. J.B. is funded by the Oxford Population Health. E.R.M., clinical lecturer, is presently supported by the NIHR and the Academy of Medical Sciences. The funders had no role in study design, data collection and analysis, decision to publish, or preparation of the manuscript.

## Author contribution

J.B.: conceptualization, formal analysis, and writing – original draft; P.S.: validation, resources, and writing – review and editing; NBSR Collaborators: data curation, funding acquisition, and resources; S.A.J.: conceptualization, supervision, and writing – review and editing; P.A.: conceptualization, supervision, and writing – review and editing; O.K.: conceptualization, validation, resources, and writing – review and editing; E.R.M.: conceptualization, formal analysis, writing – original draft, and writing – review and editing.

## Conflicts of interest disclosure

The authors declare that they have no financial conflict of interest with regard to the content of this report.

## Research registration unique identifying number (UIN)

Registered at on ClinicalTrials.gov with the identifier NCT06324526.

## Guarantor

John Buckell and Emma Rose McGlone.

## Data availability statement

The journal requires authors to include in any articles that report results derived from research data to include a Data Availability Statement. Please confirm if any datasets generated during and/or analysed during the current study are publicly available, available upon reasonable request, or if data sharing is not applicable to this article. The data that support the findings of this study are available from the corresponding author (ERM), upon reasonable request.

## Provenance and peer review

Invited.

## Supplementary Material

## References

[1] Dai H Alsalhe TA Chalghaf N . The global burden of disease attributable to high body mass index in 195 countries and territories, 1990-2017: an analysis of the Global Burden of Disease Study. PLoS Med 2020;17:e1003198. 32722671 10.1371/journal.pmed.1003198 PMC7386577

[2] Global Burden of Disease Diabetes Collaborators . Global, regional, and national burden of diabetes from 1990 to 2021, with projections of prevalence to 2050: a systematic analysis for the Global Burden of Disease Study 2021. Lancet 2023;402:203–234. 37356446 10.1016/S0140-6736(23)01301-6 PMC10364581

[3] Kivimäki M Strandberg T Pentti J . Body-mass index and risk of obesity-related complex multimorbidity: an observational multicohort study. Lancet Diabetes Endocrinol 2022;10:253–263. 35248171 10.1016/S2213-8587(22)00033-X PMC8938400

[4] Sarwer DB Polonsky HM . The psychosocial burden of obesity. Endocrinol Metab Clin North Am 2016;45:677–688. 27519139 10.1016/j.ecl.2016.04.016 PMC6052856

[5] Kolotkin RL Andersen JR . A systematic review of reviews: exploring the relationship between obesity, weight loss and health-related quality of life. Clin Obes 2017;7:273–289. 28695722 10.1111/cob.12203 PMC5600094

[6] Buckell J Mei XW Clarke P . Weight loss interventions on health-related quality of life in those with moderate to severe obesity: findings from an individual patient data meta-analysis of randomized trials. Obes Rev 2021;22:e13317. 34374197 10.1111/obr.13317

[7] Soltoft F Hammer M Kragh N . The association of body mass index and health-related quality of life in the general population: data from the 2003 Health Survey of England. Qual Life Res 2009;18:1293–1299. 19813103 10.1007/s11136-009-9541-8 PMC2788145

[8] Coulman KD Blazeby JM . Health-related quality of life in bariatric and metabolic surgery. Curr Obes Rep 2020;9:307–314. 32557356 10.1007/s13679-020-00392-z PMC7447653

[9] O’Brien PE Hindle A Brennan L . Long-term outcomes after bariatric surgery: a systematic review and meta-analysis of weight loss at 10 or more years for all bariatric procedures and a single-centre review of 20-year outcomes after adjustable gastric banding. Obes Surg 2019;29:3–14. 30293134 10.1007/s11695-018-3525-0 PMC6320354

[10] Mingrone G Panunzi S De Gaetano A . Metabolic surgery versus conventional medical therapy in patients with type 2 diabetes: 10-year follow-up of an open-label, single-centre, randomised controlled trial. Lancet 2021;397:293–304. 33485454 10.1016/S0140-6736(20)32649-0

[11] Coulman KD MacKichan F Blazeby JM . Patient experiences of outcomes of bariatric surgery: a systematic review and qualitative synthesis. Obes Rev 2017;18:547–559. 28273694 10.1111/obr.12518 PMC5709707

[12] De Luca M Zese M Bandini G . Metabolic bariatric surgery as a therapeutic option for patients with type 2 diabetes: A meta-analysis and network meta-analysis of randomized controlled trials. Diabetes, Obesity and Metabolism 2023;25:2362–2373. 10.1111/dom.15117 37272316

[13] Tan SYT Tham KW Ganguly S . The impact of bariatric surgery compared to medical therapy on health-related quality of life in subjects with obesity and type 2 diabetes mellitus. Obesity Surgery 2021;31:829–837. 33063154 10.1007/s11695-020-05038-6

[14] Magallares A Schomerus G . Mental and physical health-related quality of life in obese patients before and after bariatric surgery: A meta-analysis. Psychology, Health & Medicine 2015;20:165–176. 10.1080/13548506.2014.963627 25258028

[15] Griffiths A Paracha N Davies A . Analyzing health-related quality of life data to estimate parameters for cost-effectiveness models: an example using longitudinal EQ-5D data from the SHIFT randomized controlled trial. Adv Ther 2017;34:753–764. 28205056 10.1007/s12325-016-0471-x PMC5350196

[16] Pais-Ribeiro JL . Quality of life is a primary end-point in clinical settings. Clin Nutr 2004;23:121–130. 14757401 10.1016/s0261-5614(03)00109-2

[17] Wye L Brangan E Cameron A . Evidence based policy making and the ‘art’ of commissioning - how English healthcare commissioners access and use information and academic research in ‘real life’ decision-making: an empirical qualitative study. BMC Health Serv Res 2015;15:430. 26416368 10.1186/s12913-015-1091-x PMC4587739

[18] British Obesity and Metabolic Surgery society . *Third NBSR Report* 2020. Accessed 1 February, 2022. https://www.e-dendrite.com/NBSR2020

[19] World Medical Association . World Medical Association Declaration of Helsinki. Ethical principles for medical research involving human subjects. Bull World Health Organiz 2001;79:373–374. PMC2566407 11357217

[20] Mathew G Agha R Group S . STROCSS 2021: strengthening the reporting of cohort, cross-sectional and case-control studies in surgery. Int J Surg 2021;96:106165. 34774726 10.1016/j.ijsu.2021.106165

[21] National Institute of Clinical Excellence . Obesity: identification, assessment and management of overweight and obesity in children, young people and adults (partial update of CG43). (2014). Accessed 23 June, 2024. https://www.nice.org.uk/guidance/cg189

[22] Herdman M Gudex C Lloyd A . Development and preliminary testing of the new five-level version of EQ-5D (EQ-5D-5L). Qual Life Res 2011;20:1727–1736. 21479777 10.1007/s11136-011-9903-x PMC3220807

[23] National Institute of Clinical Excellence . Position statement on use of the EQ-5D-5L value set for England (updated October 2019). Accessed 30 June, 2023. https://www.nice.org.uk/about/what-we-do/our-programmes/nice-guidance/technology-appraisal-guidance/eq-5d-5l

[24] Mulhern B Meadows K . The construct validity and responsiveness of the EQ-5D, SF-6D and Diabetes Health Profile-18 in type 2 diabetes. Health Qual Life Outcomes 2014;12:42. 24661350 10.1186/1477-7525-12-42 PMC4304018

[25] van Hout B Janssen MF Feng YS . Interim scoring for the EQ-5D-5L: mapping the EQ-5D-5L to EQ-5D-3L value sets. Value Health Jul-Aug 2012;15:708–715. 10.1016/j.jval.2012.02.008 22867780

[26] Wood SN . Generalized Additive Models: An Introduction with R, Second Edition. Chapman and Hall/CRC; 2017.

[27] Bell A Fairbrother M Jones K . Fixed and random effects models: making an informed choice. Quality & Quantity 2019;53:1051–1074.

[28] Lindekilde N Gladstone BP Lübeck M . The impact of bariatric surgery on quality of life: a systematic review and meta-analysis. Obes Rev 2015;16:639–651. 26094664 10.1111/obr.12294

[29] Askari A Arhi C Mamidanna R . Quality of life following bariatric and metabolic surgery Athanasiou T, Patel V, Darzi A, eds. Patient Reported Outcomes and Quality of Life in Surgery. Springer International Publishing; 2023:85–96.

[30] Driscoll S Gregory DM Fardy JM . Long-term health-related quality of life in bariatric surgery patients: A systematic review and meta-analysis. Obesity (Silver Spring) 2016;24:60–70. 26638116 10.1002/oby.21322

[31] Eisenberg D Shikora SA Aarts E . 2022 American Society for Metabolic and Bariatric Surgery (ASMBS) and International Federation for the Surgery of Obesity and Metabolic Disorders (IFSO): indications for metabolic and bariatric surgery. Surg Obes Reltd Dis 2022;18:1345–1356. 10.1016/j.soard.2022.08.013 36280539

[32] By-Band-Sleeve Collaborative Group . Roux-en-Y gastric bypass, gastric banding, or sleeve gastrectomy for severe obesity: baseline data from the By-Band-Sleeve randomized controlled trial. Obesity 2023;31:1290–1299. 37140395 10.1002/oby.23746

[33] Breeze P Gray LA Thomas C . Estimating the impact of changes in weight and BMI on EQ-5D-3L: a longitudinal analysis of a behavioural group-based weight loss intervention. Qual Life Res 2022;31:3283–3292. 35796997 10.1007/s11136-022-03178-z PMC9546944

[34] Fiorani C Coles SR Kulendran M . Long-term quality of life outcomes after laparoscopic sleeve gastrectomy and Roux-en-Y Gastric bypass-a comparative study. Obes Surg 2021;31:1376–1380. 33064260 10.1007/s11695-020-05049-3 PMC7920888

[35] Minhas M Murphy CM Balodis IM . Food addiction in a large community sample of Canadian adults: prevalence and relationship with obesity, body composition, quality of life and impulsivity. Addiction 2021;116:2870–2879. 33843091 10.1111/add.15446

[36] Leahey TM Bond DS Raynor H . Effects of bariatric surgery on food cravings: do food cravings and the consumption of craved foods “normalize” after surgery? Surg Obes Relat Dis 2012;8:84–91. 21925967 10.1016/j.soard.2011.07.016 PMC4438677

[37] Al-Alsheikh AS Alabdulkader S Johnson B . Effect of obesity surgery on taste. Nutrients 2022;14:866. doi:10.3390/nu14040866 35215515 PMC8878262

[38] Graham Y Hayes C Small PK . Patient experiences of adjusting to life in the first 2 years after bariatric surgery: a qualitative study. Clinical Obesity 2017;7:323–335. 28744976 10.1111/cob.12205 PMC5763321

[39] McGlone ER Carey I Velickovic V . Bariatric surgery for patients with type 2 diabetes mellitus requiring insulin: clinical outcome and cost-effectiveness analyses. PLoS Med 2020;17:e1003228. 33285553 10.1371/journal.pmed.1003228 PMC7721482

[40] Caspersen CK de Place TB Christiansen TMB . Partial remission of Type 2 diabetes and changes in quality of life after gastric bypass. Dan Med J 2021;68:A09190484. 33543699

[41] Weiner S Neugehauer EA . Quality of life of diabetic patients with medical or surgical treatment. Nutr Hosp 2013;28(Suppl 2):66–77. 23834049 10.3305/nh.2013.28.sup2.6716

[42] Sierżantowicz R Ładny JR Lewko J . Quality of life after bariatric surgery-a systematic review. Int J Environ Res Public Health 2022;19:9078. doi:10.3390/ijerph19159078 35897447 PMC9330722

[43] Busutil R Espallardo O Torres A . The impact of obesity on health-related quality of life in Spain. Health Qual Life Outcomes 2017;15:197. 29017494 10.1186/s12955-017-0773-y PMC5634835

[44] McCarney R Warner J Iliffe S . The Hawthorne Effect: a randomised, controlled trial. BMC Med Res Methodol 2007;7:30. 17608932 10.1186/1471-2288-7-30 PMC1936999

[45] Catalogue of Biases Collaboration SE, Brassey J . Ascertainment bias. 2017. Accessed 14 June, 2024. https://catalogofbias.org/biases/ascertainment-bias/

[46] Antonsson T Wennersten A Sörensen K . Differences in health-related quality of life after gastric bypass surgery: a cross-sectional study. Obes Surg 2021;31:3194–3202. 33928524 10.1007/s11695-021-05416-8 PMC8175313

[47] Major P Stefura T Dziurowicz B . Quality of life 10 years after bariatric surgery. Obes Surg 2020;30:3675–3684. 32535784 10.1007/s11695-020-04726-7 PMC7467960

