# Supplementary material for: What is the effect of bariatric surgery on health-related quality of life in people with obesity? Observational cohort analysis of the United Kingdom National Bariatric Surgery Registry
Source: Int J Surg. 2024 Aug 22;110(11):6898–905. doi: 10.1097/JS9.0000000000002044 (PMC11573071; doi:10.1097/JS9.0000000000002044)
Supplement: Supplementary file 1 [file js9-110-6898-s001.docx]

**Supplementary Figure 1: Flow chart to illustrate inclusion of records**
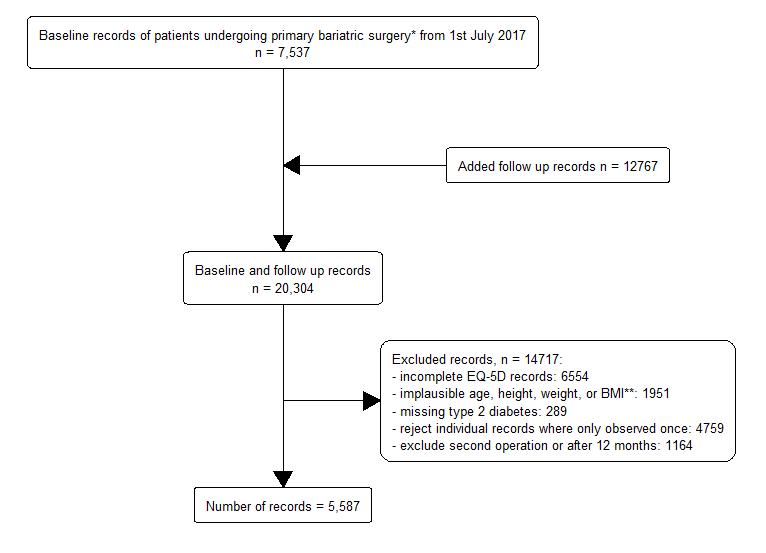


BMI = Body Mass Index

*Primary bariatric surgery: adjustable gastric band (AGB), sleeve gastrectomy (SG), Roux-en-Y gastric bypass (RYGB) and single-anastomosis gastric bypass (OAGB)

** age >100 years; height <1m or >2.5m; weight <50kg or >400kg; BMI <25 kg/m^2^ or >100 kg/m^2^

**Supplementary Table 1: number of records in data at months from baseline**

| Months from baseline | Number of records |
| --- | --- |
| 0 | 2160 |
| 1 | 654 |
| 2 | 641 |
| 3 | 396 |
| 4 | 279 |
| 5 | 269 |
| 6 | 299 |
| 7 | 208 |
| 8 | 178 |
| 9 | 132 |
| 10 | 96 |
| 11 | 68 |
| 12 | 207 |

**Supplementary Table 2: Regression outputs from models**

| Time horizon  Specification  EQ-5D  BMI |  | Model 1  Baseline  Static HRQoL, static BMI  EQ-5D(t)  BMI(t) | | | | | |  | Model 2  12 month  Change in HRQoL, change in BMI  EQ-5D(t)-EQ-5D(t-1)  BMI(t)-BMI(t-1) | | | | | |
| --- | --- | --- | --- | --- | --- | --- | --- | --- | --- | --- | --- | --- | --- | --- |
|  |  | Estimate | Std. Error | LCB | UCB | t value | Pr(>\|t\|) |  | Estimate | Std. Error | LCB | UCB | t value | Pr(>\|t\|) |
|  |  |  |  |  |  |  |  |  |  |  |  |  |  |  |
| (Intercept) |  | 0.978 | 0.180 | 0.625 | 1.332 | 5.425 | 0.000 |  | -0.021 | 0.140 | -0.296 | 0.253 | -0.151 | 0.889 |
| BMI |  | -0.005 | 0.000 | -0.006 | -0.004 | -9.977 | 0.000 |  |  |  |  |  |  |  |
| delta BMI |  |  |  |  |  |  |  |  | -0.006 | 0.001 | -0.007 | -0.005 | -9.776 | 0.000 |
| Group mean BMI |  |  |  |  |  |  |  |  | 0.001 | 0.000 | 0.000 | 0.002 | 3.143 | 0.002 |
| Male |  | 0.031 | 0.010 | 0.012 | 0.051 | 3.166 | 0.002 |  | -0.004 | 0.008 | -0.019 | 0.011 | -0.517 | 0.618 |
| Non-White |  | 0.004 | 0.012 | -0.020 | 0.027 | 0.292 | 0.771 |  | 0.004 | 0.009 | -0.014 | 0.022 | 0.400 | 0.702 |
| Employment: Not recorded |  | -0.027 | 0.023 | -0.071 | 0.017 | -1.184 | 0.236 |  | -0.008 | 0.019 | -0.045 | 0.028 | -0.450 | 0.666 |
| Employment: Retired |  | -0.066 | 0.019 | -0.103 | -0.030 | -3.582 | 0.000 |  | 0.028 | 0.014 | 0.000 | 0.055 | 1.969 | 0.049 |
| Employment: Unemployed |  | -0.090 | 0.011 | -0.111 | -0.069 | -8.486 | 0.000 |  | 0.019 | 0.008 | 0.003 | 0.035 | 2.347 | 0.019 |
| T2D |  | 0.009 | 0.010 | -0.011 | 0.029 | 0.861 | 0.389 |  | 0.002 | 0.012 | -0.022 | 0.026 | 0.195 | 0.856 |
| T2D remission |  |  |  |  |  |  |  |  | 0.037 | 0.011 | 0.015 | 0.059 | 3.253 | 0.001 |
| T2D developed |  |  |  |  |  |  |  |  | -0.015 | 0.035 | -0.083 | 0.053 | -0.431 | 0.680 |
| comorbidity count1 |  | -0.043 | 0.009 | -0.059 | -0.026 | -4.931 | 0.000 |  | -0.021 | 0.007 | -0.035 | -0.006 | -2.819 | 0.005 |
| comorbidity count2 |  | -0.105 | 0.011 | -0.127 | -0.084 | -9.501 | 0.000 |  | -0.012 | 0.011 | -0.033 | 0.009 | -1.146 | 0.255 |
| comorbidity count3 |  | -0.211 | 0.015 | -0.240 | -0.182 | -14.240 | 0.000 |  | -0.047 | 0.017 | -0.080 | -0.014 | -2.798 | 0.005 |
| comorbidity count4 |  | -0.254 | 0.025 | -0.302 | -0.206 | -10.343 | 0.000 |  | 0.005 | 0.034 | -0.062 | 0.071 | 0.134 | 0.902 |
| comorbidity count5 |  | -0.077 | 0.159 | -0.389 | 0.234 | -0.485 | 0.628 |  |  |  |  |  |  |  |
| Type of surgery: Bypass |  |  |  |  |  |  |  |  | -0.019 | 0.012 | -0.043 | 0.005 | -1.532 | 0.126 |
| Type of surgery: Sleeve |  |  |  |  |  |  |  |  | -0.004 | 0.011 | -0.026 | 0.018 | -0.342 | 0.746 |
| current age |  | -0.001 | 0.000 | -0.002 | 0.000 | -2.814 | 0.005 |  | 0.001 | 0.000 | 0.000 | 0.001 | 2.439 | 0.015 |
| IMD19 |  | 0.000 | 0.000 | 0.000 | 0.000 | 0.617 | 0.538 |  | 0.000 | 0.000 | 0.000 | 0.000 | -0.719 | 0.481 |
| days from baseline |  |  |  |  |  |  |  |  | 0.000 | 0.000 | 0.000 | 0.000 | -6.736 | 0.000 |
| ns(time cal, df = 5)1 |  | 0.132 | 0.045 | 0.043 | 0.221 | 2.913 | 0.004 |  | 0.029 | 0.048 | -0.065 | 0.123 | 0.601 | 0.559 |
| ns(time cal, df = 5)2 |  | 0.059 | 0.054 | -0.047 | 0.164 | 1.091 | 0.275 |  | 0.106 | 0.057 | -0.007 | 0.219 | 1.846 | 0.064 |
| ns(time cal, df = 5)3 |  | 0.044 | 0.032 | -0.020 | 0.107 | 1.353 | 0.176 |  | 0.048 | 0.035 | -0.020 | 0.116 | 1.396 | 0.164 |
| ns(time cal, df = 5)4 |  | 0.180 | 0.104 | -0.024 | 0.384 | 1.726 | 0.084 |  | 0.099 | 0.111 | -0.119 | 0.316 | 0.891 | 0.379 |
| ns(time cal, df = 5)5 |  | -0.025 | 0.027 | -0.079 | 0.028 | -0.921 | 0.357 |  | 0.058 | 0.021 | 0.017 | 0.099 | 2.772 | 0.006 |
|  |  |  |  |  |  |  |  |  |  |  |  |  |  |  |
| Hospital fixed effects |  | Yes | | | | | |  | Yes | | | | | |
| Individual random effects |  | No | | | | | |  | Yes | | | | | |
|  |  |  |  |  |  |  |  |  |  |  |  |  |  |  |
| Inds |  | 2160 | | | | | |  | 2160 | | | | | |
| Obs |  | 2160 | | | | | |  | 3427 | | | | | |
| AIC |  | -1784.0 | | | | | |  | -2670.19 | | | | | |
|  |  |  |  |  |  |  |  |  |  |  |  |  |  |  |

Std. Error - robust standard error, Inds – number of individuals, Obs – number of observations, LCB – lower bound of 95% confidence interval, UCB – upper bound of 95% confidence interval, t-value – t-ratio, P(>|t|) – probability of being Greater than the absolute critical value of t, AIC – Akaike Information Criterion.
